# FALLS AMONG CAREGIVERS AGE 45 AND OLDER IN 15 STATES, DC, AND PUERTO RICO, 2016

**DOI:** 10.1093/geroni/igz038.2093

**Published:** 2019-11-08

**Authors:** Erin D Bouldin, Vanna Labi, Valerie Edwards, Lisa McGuire

**Affiliations:** 1 Appalachian State University, Boone, North Carolina, United States; 2 Centers for Disease Control and Prevention, Atlanta, Georgia, United States

## Abstract

Family and friends who provide care to people with chronic conditions or disability (caregivers) are a critical component of community-based care in the US. Falls could negatively impact a caregiver’s ability to provide care. In 2016, 15 states, DC, and Puerto Rico included the optional Caregiver Module on the Behavioral Risk Factor Surveillance System. We used these data to estimate fall and injurious fall prevalence among caregivers age ≥45 who had provided care for ≥2 years. Among 71,541 respondents, 11.8% provided care for ≥2 years. One-third of caregivers had fallen in the past year (16.6% once; 16.4% more than once), and 47.3% of caregivers who fell reported at least one resulted in an injury that limited activity or required treatment. Caregivers were significantly more likely to fall (p=0.02) and to experience injury (p=0.002) compared to non-caregivers. Falls are common among caregivers and potentially harmful to them and their care recipients.

